# COVID-19 vaccination and test management for healthcare workers—development, implementation and feasibility of a custom human resources information platform at a university hospital

**DOI:** 10.1186/s12911-025-02974-0

**Published:** 2025-03-24

**Authors:** Matthias Bonigut, Ana Zhelyazkova, Mathias Weber, Stefanie Geiser-Metz, Markus Geis, Bernhard Heindl, Stephan Prückner

**Affiliations:** 1 Institut für Notfallmedizin und Medizinmanagement (INM), LMU Klinikum, Schillerstr, 53, 80336 Munich, Germany; 2 Department of Strategic Business Development, LMU Klinikum, Marchioninistr, 15, 81377 Munich, Germany

**Keywords:** COVID-19, Hospital management, Healthcare workforce, Pandemic response, Data security, Data interoperability, Data administration

## Abstract

**Background:**

The continuously evolving legislative and reporting requirements during the COVID-19 pandemic posed the demand for establishing an efficient real-time human resources management system at the LMU University Hospital, one of the largest university hospitals in Germany. Developing a system allowing for agile real-time analysis as well as for reporting employees’ COVID-19 vaccination and testing status while ensuring the security of personnel data presented several technical and managerial challenges.

**Methods:**

We designed and implemented a custom COVID-19 human resources information platform in order to fulfill the LMU University Hospital’s legal requirement to report employees’ vaccination and testing status. We designed the platform as an all-in-one solution for all relevant COVID-19 data, merged from five individual sources. The development process was guided by the principles of findability, accessibility, interoperability and reusability (FAIR) with particular focus on interoperability. Here, we present the platform’s design, cumulative user data and discuss the feasibility of the approach including its intended and unintended outcomes.

**Results:**

The COVID-19 human resources management platform was the first solution of its kind at the LMU University Hospital, emerging from the specific need for an efficient exterior and interior mandate fulfillment. It served both for *operational management* purposes as well as for *strategic pandemic and hospital management*. The immediate dependency on data privacy and regulatory adaptations due to the evolving pandemic situation posed the necessity for regular adaptations to the platform’s structure.

**Conclusions:**

The presented case reveals how data utilization requires the concurrent and proactive consideration of data security and interoperability against the background of a scalable architecture. Simultaneously, the development of such platforms needs to be open to new cases, functions and sources, thus requiring a dynamic and agile environment.

## Background

As one of the largest university hospitals in Germany, the LMU University Hospital faced several acute organizational challenges during the COVID-19 pandemic [1, 2]. Among these was the immediate establishment of an internal vaccination and test centers for all employees following the approval of the first COVID-19 vaccines [3, 4].

The vaccine shortages at the beginning of the vaccination campaign as well as the legally binding prioritization of high-risk population and employment groups postulated the detailed tracking of vaccine dose allocation [5, 6]. Following the adoption of the amendment to the Prevention Protection Act on December 11, 2021, the facility-based COVID-19 vaccination mandate in the health care sector was established and, consequently, the mandatory collection and reporting of the COVID-19 vaccination status of employees on the part of their employers was statutorily regulated [7]. These legislative changes added to the already existing requirement for regular testing and reporting of healthcare workers’ (HCWs) COVID-19 test results [8].

The reporting requirements for HCWs‘ vaccination and testing status presented specific and urgent administrative and technical challenges for the LMU University Hospital. The heterogeneous vaccination and testing services, i.e., both at the hospital and through external providers such as municipal vaccination and testing centers, complicated the data collection. Furthermore, LMU University Hospital lacked a uniform system that would allow both structured data collection and automated reporting within the hospital (i.e., to the occupational medical service) and outside the hospital (i.e., to the health department). An extension of the already implemented human resources system (SAP HR) at the hospital was technically and legally not possible, since the LMU University Hospital, as an employer, was requested as per the General Data Protection Regulation to collect and manage health and human resources data separately These circumstances revealed the need for the development and implementation of a custom in-house system for structured and privacy-compliant data collection on the COVID-19 status of employees and for automated and secure reporting to the health department.

This article presents the organizational and technical development and implementation of a custom interactive platform for structured and scalable collection and processing of COVID-19-related data of employees of the LMU University Hospital. Furthermore, we also reflect on relevant lessons learned and outline recommendations for the development of comparable human resources (HR) platforms in similar contexts.

At the time of submission of this work, the authors are not aware of other publications presenting the technical implementation of the COVID-19 status report mandate in German healthcare facilities. The requirements of the LMU University Hospital’s case reflect the issues to be considered by HR professionals when operationalizing legal COVID-19 vaccination and testing mandates outlined by Tursunbayeva et al., yet reports on the application of tools on the organizational level remain unavailable [9]. Electronic health records of HCWs and applications for nosocomial contact-tracing and vaccination status report have been previously described as individual solutions, however these are limited in scope and scale compared to the requirements and implementation of the LMU University Hospital’s case [10, 11].

A national survey on the implementation of key infection prevention and control structures in German hospitals reported that 84.9% of hospitals had informatics/IT support to conduct their surveillance (e.g. equipment, mobile technologies, electronic health records) in 2019 and 89.4% did in 2023, however the survey did not provide information on the utilized tools [12, 13].

## Methods

We outline the design and functionalities of the platform as well as the adjustments applied to it. The resulting intended and unintended effects are presented and discussed using Parry and Tyson’s framework [14].

Further, we present relevant usage data as of 31.12.2022, which corresponds to the status of the platform prior to the deletion of the primary data and the irreversible anonymization based on it. The anonymization process entailed the irreversible deletion of all identifiers, e.g. personal HR number, names, date of birth. The development and usage of the platform including for scientific purposes were approved by the Data Security Officer (Project number: 1830.1) and audited by the Bavarian Data Protection Commissioner. Hospitals were legally bound to collect and report all COVID-19 vaccination and testing data of persons working in their facilities, thus waiving the prerequisite of individual consent [8, 15]. However, employees were able to request a deletion of their COVID-19 vaccination data from the platform. Ethical approval and consent for the data and analyses presented here were waived in view of the retrospective nature of this project and the use of solely irreversibly anonymized data (project number waiver: 23–0284 KB).

### Development of the platform

The team of the Institute of Emergency Medicine and Medical Management (INM) was tasked with the development, implementation, technical and operational support of the platform. The application was developed in Oracle Application Express (APEX 21.1.1.) with the following requirements:

- Entering, storing and updating employees’ data on:
  - COVID-19 vaccination status including date and vaccine type;
  - COVID-19 test results (negative as well as positive, within the framework of regular testing as well as also in the case of testing to confirm negativity following an infection);
- Digitalization and automation of the test management including reporting of positive test results by the in-hospital laboratory to the person tested (by telephone, e-mail), their manager, the occupational medical service and the municipal health department;
- Compilation of interactive statistics for epidemiological and staff monitoring of pandemic effects on the hospital.

Due to the high complexity and relevance of the task, the design and development process was guided by the FAIR principles - findability, accessibility, interoperability and reusability [16].

The target groups of the defined users were limited to the hospital’s internal departments and employees in management positions. Based on the distribution of their tasks, the users can be divided into two main categories: **strategic pandemic and hospital management** (HR department, pandemic officers, and occupational medical services) (1) and **operational management** (employees in management positions) (2). For users in target group 1, the interactive data evaluation functions of the platform were defined as a monitoring tool for outbreak management and as a basis for strategic decision-making processes. For target group 2, personnel management at the team level was defined as the main task, including data quality management, entering new data and correcting false or outdated information (e.g. in the case of external positive testing). The task management, especially for target group 2, was derived from the legislative obligation for COVID-19 reporting [8].

### Data security and protection

The project’s data were stored on an Oracle Real Application Cluster (19.8), thus all data could be accessed from similar databases. The systems were located behind a firewall in a database zone and are heavily secured. A full backup was executed automatically every six hours with an additional backup for every single transaction including point-in-time recovery. The user administration is established via a Lightweight Directory Access Protocol (LDAP; Oracle-Unified-Directory).

Access to the data was available only to the trained administrators and developers of INM (3 persons in total). Access authorizations were granted on a department-specific basis (target group 2) – the specific users to receive access were identified by the Department of Medical Organizational Development. Each user could only view the employees assigned to them in their respective unit using their individual LDAP account login – units were defined as either wards, departments, institutes, academic chairs or other team constellations and one or more user groups could be assigned to the one users.

The Virtual Private Database (VPD) function was based on four elements: area, service type, organizational unit, occupational group. The users were located in the LDAP directory of INM in their own LDAP organization unit and were activated specifically and only for this platform.

The hierarchical organization of the users was further applied to the communication activities of and towards the platform. Data management requests were sent solely to users of target group 2, whereas users of target group 1 were only provided with cumulative data excluding any personal information of the employees included in the respective report. This meant that target group 2 users were able to view and edit all employee data, including personal identifiers, while target group 1 were able to view only metadata, e.g. number of currently infected employees in a department or of not vaccinated employees. This dynamic anonymization process based on the user’s role and group, i.e. their LDAP account, regulated the data access and allowed for the daily and secure reporting of data that were subsequently implemented in the decision-making processes of the hospital’s strategic pandemic management.

## Results

**Fig. 1:**
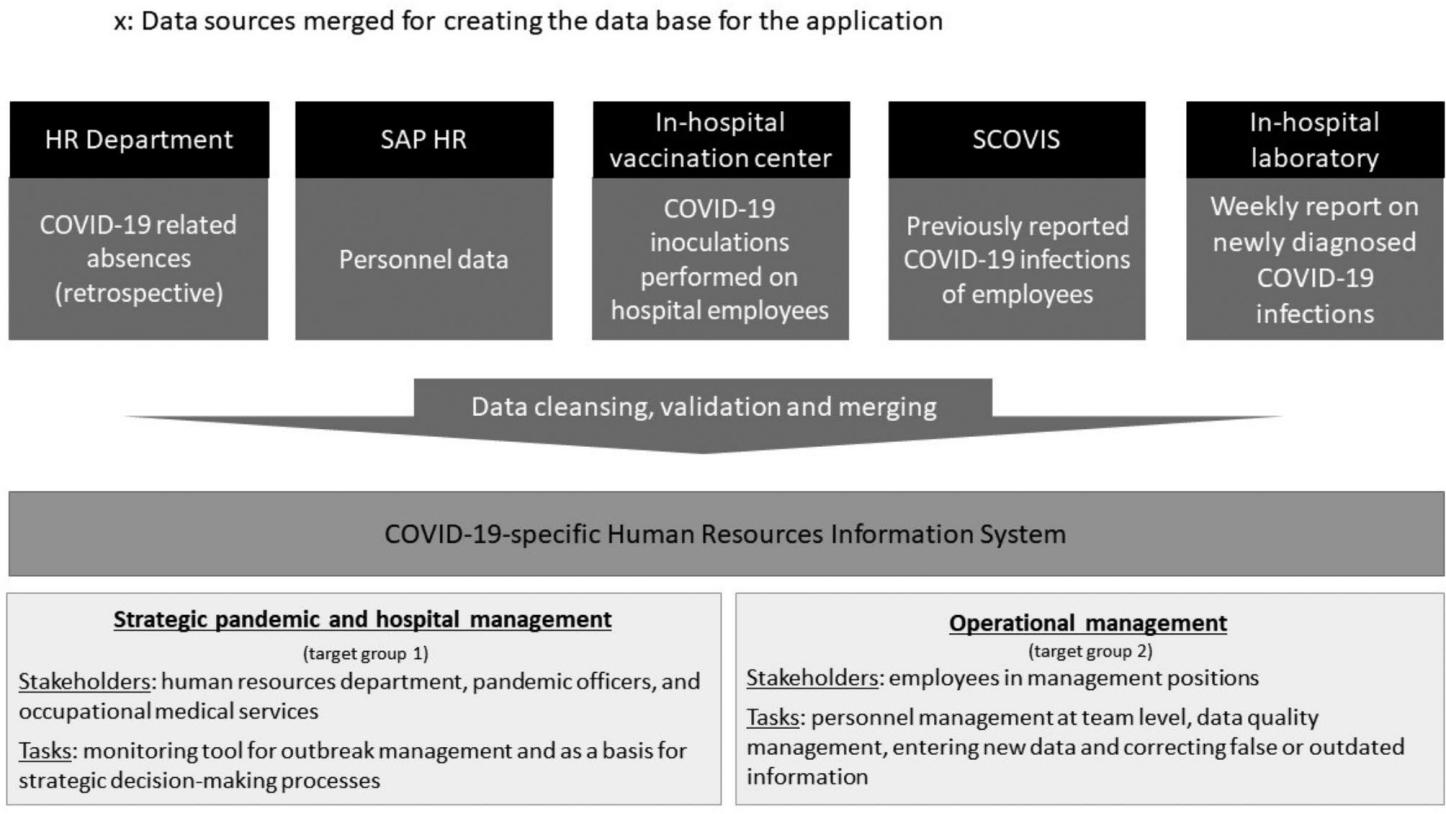
Simplified structure of the platform‘s database sources and target groups

The platform was developed as a COVID-19-specific Human Resources Information System (HRIS) and operated between November 2021 and December 2022 [17]. Five in-hospital sources of data contributed to the information basis of the platform (Fig. 1):

1. The **SAP HR** system provided all relevant personnel data that were subsequently complemented by the further four sources;
2. The **vaccination center database** provided data for all inoculations of employees carried out on-site including the vaccine type, sequence and date;
3. A further database launched as an interim solution at the beginning of the pandemic for reporting positively tested employees (**SCOVIS**) provided data on the employees that had already been infected;
4. The **HR** department retrospectively imported all previously collected data on COVID-19 absences due to positive testing or quarantine;
5. Lastly, the platform received weekly updates from the **in-hospital laboratory** on the positively tested employees, which were further manually entered by the occupational medical service.

Upon merging, all data were cleaned, validated for uniformity between the sources and corrected, if needed. The merger of these five previously independent data sources emphasized the topic of interoperability as a priority in the process, as main issues consisted in ensuring that HR personal numbers were indeed unique, other identifiers (e.g. name, date of birth) were correctly imported or corrected upon encountering false or invalid entries in any of the databases.

The responsibilities for the management of individual employee data were allocated from the HR department to the respective direct managers (target group 2). The decentralization of data management was planned and prompted as a measure to improve data quality monitoring and accelerate the feedback loop in cases of data correction demands.

For the architecture of the platform to correctly reflect the HR structure, the external subcontractors were assigned the respective rights for viewing and editing the COVID-19-related data of their direct team members through a secured interface without direct login into the system, following an additional upload of the information on external employees. This constitutes a diversion from the standard procedures at the hospital, as external employees and subcontractors do not usually have credentials for the hospital’s system.

A similar challenge was presented by the curriculum of the students of the medical faculty and the hospital’s nursing school, as these were also legally considered employees for the period of their internships. As students would regularly change their department depending on their internship rotation plan, assigning them to one department was not an efficient option as they would leave the respective team as scheduled. Hence, we created an additional “peripheral” department in order to catalogue the data of all employees that are otherwise not assigned to one specific unit.

### Timeline and functionality updates

**Fig. 2:**
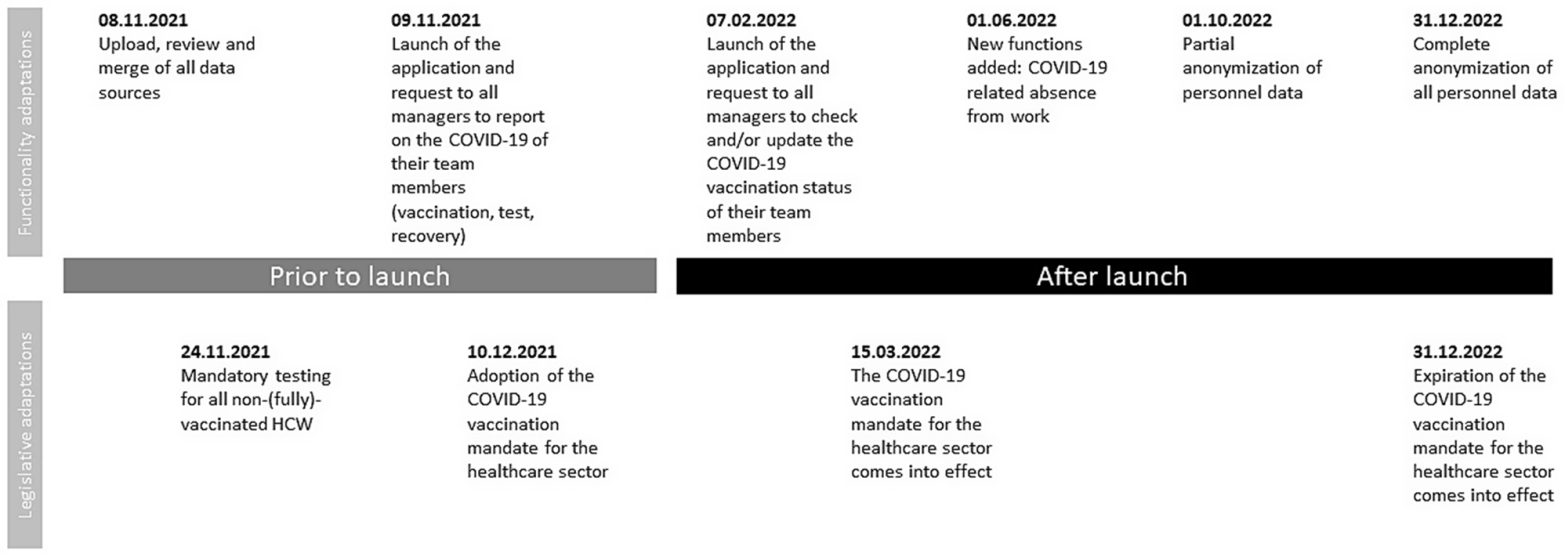
Timeline of the development and launch of the platform including legislative adaptations

The design of the platform was based on the need to collect and report data on the COVID-19 status of employees in terms of their vaccination, infection and recovery status (abbreviated in German as “3G”). Several legislative changes in the pandemic management on local, regional and country level adopted throughout 2022 triggered functional adaptations (Fig. 2).

### Types of data

We adapted the HRIS abstraction tool developed by Riley et al. in order to facilitate the comparison to other platforms [18] (Table 1). For each individual employee, we collected, merged and updated a set of data consisting of three main categories: core personnel data, 3G status and reason for absence (Table 2).

**Table 1:** Platform characteristics summarized using the HRIS abstraction tool; adapted from Riley et al. [18]

Platform components		Yes	No	Partially
Data collection	The system collects HRH Supply data (regulatory board, census, labor force survey, etc.).	☐	☒	☐
	The system provides data on health worker qualification/credentialing.	☒	☐	☐
	The system provides demographic data.	☒	☐	☐
	The system collects HRH demand data (e.g. employment records, health facility surveys).	☐	☒	☐
	The system provides deployment data by health facility.	☒	☐	☐
	The system provides deployment data by service area.	☒	☐	☐
	The system provides attrition data by category.	☐	☒	☐
	The system provides data on more than one type of employer (e.g. private, public, or faith-based).	☐	☒	☐
	The system links to payroll data.	☐	☒	☐
Data management	The system links to the health management information system.	☐	☒	☐
	HRH data are routinely assessed and cleaned for data accuracy and missing data fields (i.e. data validation).	☒	☐	☐
	HRH data are updated at least once a year.*	☒	☐	☐
	HRH data are linked between supply and demand.	☐	☒	☐
	HRH data are synthesized in written material that is disseminated to end users.	☒	☐	☐
Data utilization	Supply data is used to identify current licensed practitioners.	☐	☒	☐
	Data users produce data analysis and meet to discuss findings.	☒	☐	☐
	Demand data has been used to influence deployment practices (e.g. distribution, promotion, etc.)	☒	☐	☐
	Using data from the system for HR planning and/or evaluation of HR interventions.	☒	☐	☐
	There is an identified person/unit responsible for analyzing HR data.	☒	☐	☐
Sustainability and ownership	The system is partially or fully locally owned and managed. **	☒	☐	☐
	The system is endorsed by MOH and/or regulatory boards.	☒	☐	☐

*The data were automatically updated and synced three times a day (every 8 h)

**The system and all collected data are fully locally owned

**Table 2:** Constellation of the individual dataset created for each employee

Category	Variable	Type
Core personnel data	Personal HR number	Number
	Title/salutation	Nominal
	First name	String
	Last name	String
	Date of birth	Date
	Occupational group	Nominal
	Date of entry	Date
	Organizational unit	Number
	Status of activity	Nominal
	Data source	Nominal
	Organizational unit number	Number
	Street	Nominal
	Postal code	Number
	Place of residence	Nominal
	E-Mail	Nominal
	Mobile phone	Number
	Biological sex	Number
	Entrance date	Date
	Leaving date	Date
3G status – vaccination*	SputnikV	Binary
	CoronaVac	Binary
	Novavax	Binary
	Spikevax	Binary
	Comirnaty	Binary
	Janssen	Binary
	Vaxzevria	Binary
	Unknown	Binary
3G status - infection and recovery**	Positive PCR test	Binary
	Negative PCR test	Binary
	Negative Rapid Antigen Test	Binary
	Positive Rapid Antigen Test	Binary
	PCR test upon work re-entry with viral load > 1.000.000	Binary
	Recovered, negative PCR test upon work re-entry or positive PCR test with viral load < 1.000.000	Binary
Absence	Course of treatment	Binary
	Home office	Binary
	Contact person***	Nominal
	In quarantine without home office****	Nominal
	In quarantine with home office****	Nominal
	In isolation	Binary
	Sick leave	Binary
	Vacation	Binary
	Parental leave, pregnancy, breastfeeding	Binary
	Other	Binary

* For each vaccination the following data were collected: Date of inoculation, Serial number; Vaccination were aggregated to each personal file

** For each individual test the following data were collected: Date of test, Result (positive/negative); Viral load (in-hospital PCR tests only)

***Items: category 1 - <1.5 m, > 15 min; category 2 - >1.5 m or < 15 min; category 3 – other

****Items: Travel-related quarantine; Travel-related quarantine (re-entry to Germany from a high-risk area); Travel-related quarantine (re-entry to Germany from an area of variant(s) of concern)

### Data collection and management

Our platform collected demographic and deployment data in order to allow for the report of unit-centered outbreaks as well as for the decision-making process on personnel re- and allocation.

The data management prioritized the validity, accuracy and timely delivery of data. The complete dataset was updated three times a day (every eight hours). Further, the closed-loop communication facilitated a continuous feedback between the decentralized operational data management and the centralized visualization for the hospital’s strategic management, thus enabling live data-driven decision-making support.

Data users of target group 1 were regularly meeting within the frames of the Executive Board Task Force and Pandemic Board to discuss the latest data and its implications for the functioning of the hospital – details on the management structure and decision-making processes are described elsewhere [2]. The production of the data analysis as well as its presentation during the meetings was assigned to the team of INM. Both the established and regularly presented real-time analyses as well as specifically demanded and executed data inquiries were applied in the decision-making processes for deployment or relocation of personnel.

### Sustainability and ownership

The platform and all collected data were fully locally owned and managed. Due to the specifics of the data protection requirements as well as the recurring short-term adaptations of the data structure, a non-local ownership model would not have been possible. As the platform was made necessary due to the reporting requirements set by the health authorities on national, regional and local level, it was also accepted by the health authorities.

### Data volume

By 31.12.2022, the final day of the platform’s operational utilization, the databank contained 14,419 individual active files of HCWs employed at the LMU University Hospital including students and persons working at the hospital but employed through a subcontracting company (e.g. hygienic personnel). Counting the deactivated entries (e.g. staff leaving the hospital), the platform contained overall 16,220 personnel files. The platform provided overall 656 accounts of which 16 were assigned to target group 1 users and the remaining 640 accounts were attributed to target group 2 users.

**Fig. 3:**
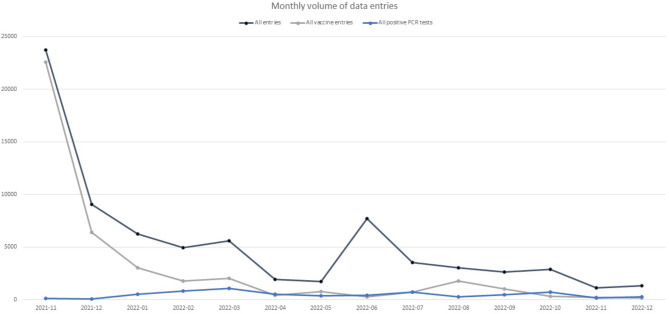
Monthly sum of data entries compared to the sums of vaccination and positive PCR test entries. The numbers presented here do not include other entries to the system, such as data on antigen tests

The monthly sum of data entries was driven mostly by the number of vaccine-related entries, where the majority of data were entered in the first month of operation (Fig. 3). An exemption to the approach can be observed in the month of June 2022, when retrospective data of all previously recorded COVID-19-related absences prior to November 2021 were imported into the system.

### Desired goals and actual outcomes

The need-based development of the platform has driven the realization of some targeted as well as untargeted changes. These are presented below based on Parry and Tyson’s framework [14] (Table 3).

**Table 3:** Comparing the intended and actual benefits of the development and implementation of the platform, adapted from Parry and Tyson [14]

Goals	Objectives and intended benefits	Operational impact and actual benefits
Operational efficiency	Fulfillment of statutory reporting requirements	Automation of the reporting process for internal users as well as for external interfaces
Service delivery	Reduction of the necessary personnel capacities as well as the time delay between event (test/vaccination) and corresponding notification.	Optimization of the applied resources within the reporting procedures as well as the accuracy of the collected, stored and reported data
Strategic orientation	Enable up-to-the-minute and interactive personnel-related assessment of the COVID-19 situation	Provide an automated and multi-layered overview of COVID-19 vaccination and testing activities among personnel.
Manager empowerment	Decentralization of reporting procedures and data management	Successfully reform the allocation of responsibility for data management from centralized to decentralized; enable direct feedback and efficient monitoring of data accuracy.
Standardization	Standardization of the reporting process for COVID-19 vaccinations and test results	Automated and up-to-date maintenance of COVID-19 data
Organizational image	-	Promoting the digitization of internal processes at the hospital

#### Operational efficiency

The implementation of the platform had the immediate effect of reducing the resources needed for the reporting processes. These were centralized in the authority of the occupational medical services, thus enabling a single point of contact for the external health authorities as well as for the affected employees.

#### Strategic orientation

The interactive report provided decision-makers (target group 1) with the opportunity for a daily overview of infection events in the hospital. The platform allowed for reporting on data queries on several organizational levels – from single units to whole clinical departments. This provided the basis for short- and long-term decisions concerning the immediate organization of capacity planning and human resources allocation especially regarding patient care and with particular focus on patient safety.

#### Manager empowerment

The decentralization on team level allowed for the direct supervision of the quality of data by the immediate managers of all employees by providing an efficient distribution of responsibilities. Further, the insight into the COVID-19-related data of employees allowed for managers to seek direct contact to team members in cases of unclear “3G” status. This also allowed for a direct feedback loop between the managers on-site and the platform team.

#### Standardization

The entries in need of correction during the standardization process were identified and corrected via notifications to and from the respective managers. The correction process resulted in the adjustment of the corresponding entries in SAP HR, thus contributing significantly to the accuracy and standardization of the initial database for personnel at the hospital.

#### Organizational image

Changes in organizational perception were not defined as a goal of the platform. Nevertheless, they had an impact on the general strategic perception of future digitization possibilities at the hospital.

#### Challenges of and during the implementation process

The process of the platform’s implementation was accompanied by some challenges. Beginning with the identification of the need to correct the database, a fundamental review of the accuracy of the entries was required. Post launch, there were daily reports of incorrect or outdated entries that had to be corrected manually as well as frequent cases of users losing their login data. The considerable resource expenditure associated with this was not taken into account at the beginning of the project. Additionally, the resources required for the daily user management, referred to by the team as “hotline”, were not taken into account in the planning phase: this includes, above all, the support of user inquiries regarding the platform’s functionalities, access data and general process organization.

## Discussion

In this article, we presented the development and implementation of a COVID-19-specific HRIS-based platform at the LMU University Hospital. The platform triggered some intended and unintended effects, in particular, the successful decentralization of data. While it was initially created as an operational application, it successfully contributed to the strategic alignment with the hospital’s pandemic management [19]. The agility of the team as well as of the technical foundation were challenged by several rounds of development build induced by changes of the legislative framework the platform was based on or adaptations to the operational and managerial necessities of the hospital. Although all data were anonymized by the end of 2022, the case of the platform provides evidence for the advantages of the implementation of similar systems at the LMU University hospital or within comparable organizations with a broader application spectrum [20].

This work contributes to the literature on HRIS-based solutions in the healthcare sector [17]. The exhaustive description of the process contributes to better understanding and exemplifying the resources and measures needed for data cleaning and quality control both prior to as well as following the launch of a HRIS-based solution [18]. The automation and efficiency achieved through the platform are particularly relevant to the targeted and met outcome of enabling evidence-based decision-making processes for stakeholders at the hospital, which referred both the organization of HCWs as well as to the management of patient care delivery for COVID- and non-COVID-19 wards [18]. Additionally, the shortened feedback loops between the platform’s team and the users allowed for a direct needs-based communication. These developments are in line with the trends observed in the implementation of HR systems in the healthcare sector [21].

Our experience shares similarities to the operations in other settings. Negro-Calduch et al. report in their international survey on the operational management of Health Information Systems adopted in 19 countries, where the main challenges likewise arise from the interoperability and communication of as well as from the need for multiple adjustments to the established or new systems developed for COVID-19 surveillance mostly in order to ensure a valid and secure decision-making foundation for stakeholders [22]. Additionally, platform designs similar to the one described here can be further enriched by implementing artificial intelligence in order to further facilitate the decision-making automation [23].

As recent research has demonstrated, the manner and degrees of freedom of hospital management are directly linked to the values intertwined with the public policies in the respective region [24]. These, in turn, affect the perception of healthcare personnel regarding the organization and managerial autonomy they have over their work [24]. Our findings additionally underline the interchangeable and complex relationship between legislative guidelines and the provision of high-quality healthcare.

### Placing the platform among the health data efforts on European level

Observing the topic from the broader perspective, tools such as our platform position themselves atop the future healthcare agenda by addressing global challenges in a local context. This pertains particularly to the establishment of a European vaccination ecosystem due to several reasons [25]. On a smaller scale and for the specific but crucial case of HCWs, our platform contributes to the monitoring of the vaccination uptake and coverage, which can be more specifically utilized to identify demands for action on the procurement, logistics and distribution of vaccinations [25]. This further allows for the monitoring of vaccine hesitancy among the targeted population [26]. Similarly, the regular short-term changes to the platform highlight the need for synchronization of legal, administrative and operational standards that should be considered when planning and developing a system of any scale [27]. These aspects ascribe to the recommendations of the European Health Management Association as well as of the Digital Health Society and the European Institute for Innovation through Health Data especially to the suggestions for consultations with health managers, accessibility and interoperability of data, implementation of standards for scalability and skills development on the road towards the European Health Data Space [25, 28, 29]. Effectively, the expected and unexpected outcomes of the implementation of the platform ascribe to the benefits of digitalization of the European healthcare strategy identified by Odone et al. in terms of improved accuracy, usability of data and automation [30].

Beyond the scope of the COVID-19 pandemic, the question remains, whether and how the positive effects of the platform, especially the facilitated digitalization and management empowerment, could be sustainably implemented into the general management of the university hospital. Louredo et al. highlight the challenge of facilitating implementation beyond a momentous case and towards a sustainable employment, where the challenge prevails specifically within university hospitals due to their complex and interconnected organizational and personnel structure [31]. As the clear accountability and role distribution as well as the provision of technical support have been previously identified as key facilitators in the sustainable implementation of measures, the continuation of the achieved goals and their long-term effect remain uncertain [32]. As for infection prevention and control beyond a pandemic situation, the majority of mandates related to employees’ health are being fulfilled during the onboarding process and do not require continuous surveillance nor additional measures demanding any actions from the employer instead of the respective laboratory (e.g. measles vaccine mandate), thus posing the question of whether a use case of pandemic or similar proportion could come to be [33]. Additionally, although Zingg et al. report on the beneficial effect of hospitals’ participation in national surveillance programs on their nosocomial infection rates, the low prioritization of infection control, restricted human resources for surveillance, as well as the lack of surveillance programs in some countries impede EU-wide expansion of this or similar efforts [34]. Lastly, considering additional HR tasks, there are currently no legislative mandates requiring a similar reporting mechanism for employers in the healthcare sector. Should the necessity arise, the irreversibly anonymized data remain at disposal for research and other previously approved purposes.

### Recommendation for HRIS-based platforms

#### Active consideration of interoperability and scalability in the development process

Data interoperability should be considered in the development process. This entails both data sources within the facility of implementation as well as potential or planned external data exchange sources. This further applies to scalability options, especially since personnel and health datasets may change or expand rapidly. Thus, the usefulness and functioning of such platform require a certain dynamic and demand-based openness to new cases, uses and user groups.

An imperative aspect to be considered throughout data integration processes is guaranteeing the data privacy and security. Specifically, two levels of this issue are to be continuously examined: secured storage and protected access.

#### Usability presumes usefulness

Acknowledging the needs of the end-users presupposes the practicality and usefulness of the platform. Although the case presented here arose from a legislative change, the need within the hospital seconded the demand for the platform as a tool for monitoring on all facility levels. This further strengthened the arguments for diligently managing the respective data as it served crucial strategic purposes for the hospital’s operations.

#### Decentralized collection and centralized management

Establishing decentralized collection with the objective of centralized management presumes the need for interoperability, standardization and accessibility of data. Simultaneously, this approach facilitates the identification of strategic potential for improvement by allowing an exhaustive overview of the data. This allows for both an operational and strategical feedback loop between the organizational levels and actors. Further, this approach strengthens the ability for planning and organizing human resources against an uncertainty-driven background, which has been established as a key managerial competency for organizational leadership to adopt during COVID-19.

#### Considering technical support necessities of end users

Inadequate staff resourcing is among the critical barriers to the sustainable adoption of hospital-based interventions [32]. Observing this recommendation from a broader long-term perspective, developing the skills of HCWs to operate confidently with digital tools is essential to the future of patient-centered healthcare and the adoption of innovative technologies in the healthcare provision [35, 36].

### Limitations

Our article and the described characteristics of the platform need to be considered against the background of several limitations. In regard to the design of this publication, we acknowledge that a survey among the users would have been beneficial and highly informative on the topic of the platform’s usability and feasibility as well as for identifying the specific barriers and facilitators to sustainably adopting the platform, potentially in an adapted form, in the HR management of the LMU University hospital. We therefore recommend an evaluation phase be planned prior to the launch of such technical measures in order to allow for their structured evaluation.

Furthermore, the platform and its dataset present several limiting aspects. In terms of testing and testing results, negative test results were not systematically imported into the platform as these were not subsequently relevant to the immediate personnel and hospital management. The only exception hereto is represented by the negative test following a previous positive test by the respective employee as this served as a clearance for re-entering the workforce. Additionally and due to the broad possibility of testing outside of the premises of the LMU University hospital, we did not have access to any data on negatively tested employees outside of the hospital premises as these did not enforce a change to their working ability. A similar challenge refers to the vaccination status data of employees, as few cases personally required the immediate deletion of their data in spite of their status. All limitations of the data pose the general question of the interaction between data security and data availability.

## Conclusions

The here presented design and implementation of a custom COVID-19 specific human resources information platform for the LMU University Hospital emphasize how an acute requirement may prompt the fulfillment of previously unidentified yet substantial operational and strategical needs. Although the essential data privacy and legislative specifications challenged both the architectural constellation and the subsequently necessary adaptations, our solution highlights the features and benefits of a decentralized operational data management with concurrent centralized strategic management.

The interoperability of data as well as the scalability of tools, platforms and applications are crucial aspects to be considered in the planning phase of digital tools and measures. These may represent a challenge even when the implementation is planned solely for a single organization. HRIS-based solutions need an agile architecture in order to provide for their adaptability to rapidly changing requirements. This further allows for the real-time, systematic and user-friendly provision of data from several sources and enables the strategic and data-driven management of personnel.

## Data Availability

The datasets generated and/or analyzed during the current study are not publicly available due data privacy and security restrictions of the LMU University Hospital but are available in cumulative form from the corresponding author on reasonable request.

## Declarations

### Author’s information

Two abstracts on the topic of this paper have been accepted for pitch presentations at the 16th European Public Health Conference, where the presentations were held by the two first authors of this publication. The accepted abstracts can be found here: https://academic.oup.com/eurpub/article/33/Supplement_2/ckad160.579/7327173; https://academic.oup.com/eurpub/article/33/Supplement_2/ckad160.411/7327419
